# Social media in undergraduate teaching and learning: A scoping review protocol

**DOI:** 10.1371/journal.pone.0291306

**Published:** 2023-11-28

**Authors:** Richard Hayman, Erika E. Smith

**Affiliations:** 1 University Library, Mount Royal University, Calgary, Alberta, Canada; 2 Academic Development Centre, Mount Royal University, Calgary, Alberta, Canada; University of Oxford, UNITED KINGDOM

## Abstract

**Objective:**

To conduct a scoping review that systematically examines the body of research on social media in undergraduate teaching and learning in order to identify key issues, trends, gaps, and needs. Our objectives include determining what methods have been commonly used to study social media in undergraduate teaching and learning, and to synthesise insights from published research findings within the fields of higher education, educational technology, and the scholarship of teaching and learning.

**Introduction:**

The use of social media technologies in post-secondary environments has been increasing over time, and especially following the shift to remote teaching and learning during the COVID-19 pandemic, this growth has continued. This review addresses a need to analyse and understand the body of research on the use of social media across undergraduate contexts for teaching and learning.

**Inclusion criteria:**

This scoping review includes peer-reviewed journal articles on social media in an undergraduate teaching or learning context published at any time, in English. In addition to including concepts and terms related to social media broadly, based on global social media usage, we include within our search the most commonly used social media platforms. We excluded items from the grey literature (such as reports, dissertations, and theses), and studies that focus on groups outside of the undergraduate population of interest (e.g., in elementary, secondary, or graduate settings, etc.).

**Methods:**

Systematic searching will be conducted in relevant subject and multidisciplinary databases: Education Database, Education Research Complete, ERIC, British Education Index, Australian Education Index, Academic Search Complete, and Scopus. Records will be deduplicated and screened using Covidence software, with each record independently reviewed by two researchers in both rounds, screening titles and abstracts in the first round, and full-text of articles in the second. Researchers will meet to discuss discrepancies and make decisions using a consensus model, and a third researcher will be independently tasked with resolving any conflicts. Data extraction will also use two independent researchers to review each article.

## Introduction and rationale

There has been an observable increase in the use of social media in post-secondary teaching and learning situations over time, and the ways these social media technologies are used in teaching and learning have become particularly relevant since the shift to remote education during the COVID-19 pandemic [1]. This scoping review research study addresses a need to analyse and understand the body of research on the use of social media across undergraduate contexts for teaching and learning. We will use a scoping review methodology to fill this gap by systematically examining approaches to studying social media in undergraduate teaching and learning, and to identify any notable patterns and themes across the findings from published studies.

There are a number of scoping reviews on contemporary educational technology issues, including digital game-based learning [2], augmented reality [3], ubiquitous learning environments [4], teen use of social media in high school [5], and academics’ use of social media [6]. However, our detailed search of the literature reveals that there are no scoping reviews on social media use focusing on teaching and learning across undergraduate contexts, published either within higher education or in the wider body of educational research. While Katz and Nandi [7] recently published a scoping review of social media in health contexts for comparison, their research focuses primarily on trainees, clinicians, and educators specifically within medical education programs during the COVID-19 pandemic. Thus, there is a clear need for a scoping study examining the topic of social media in undergraduate teaching and learning more broadly.

Although several scholars acknowledge a need to grow the field’s meta-research contributions through higher education scoping activities, issues identified with existing higher education reviews include use of problematic research design elements, focusing on overly narrow scope, and a lack of rigour in reporting [8]. Notably, Chick et al. strongly critique the search strategies and reporting standards of several recently published higher education reviews–including work by Booth and Woollacott [9] and Tight [10]–highlighting the lack of “detailed or rigorous descriptions of the review methodology” [8 p187] necessary for such studies.

Being aware of these issues and working intentionally to address them in our research, we will integrate relevant recommendations from Chick et al.’s [8] scoping review protocol where appropriate to ensure rigour in our own study while balancing for breadth and depth in our research design and reporting. We plan to share insights and examples from conducting our scoping review at a meta-research level (i.e., discussing the research *process* as well as the outcomes of our scholarship) in order to help promote further understanding and use of review methods by future higher education scholars.

Unlike other types of reviews, such as meta-analyses, a scoping review methodology embeds a systematic approach but intends to address broader topics where many different research designs and approaches have been taken. Following from Arksey and O’Malley’s foundational framework for scoping reviews, we have selected a scoping review because our research well aligns with three common reasons for conducting a scoping study: “to examine the extent, range and nature of research activity”; “to summarize and disseminate research findings”; and, “to identify research gaps in the existing literature” [11 p21].

There is growing recognition in the wider research community of the value that reviews contribute. Onwuegbuzie, Leech, and Collins [12] highlight the benefit of reviews for informing practice and understanding the topic being explored. By conducting reviews, scholars can identify trends and opportunities for using and improving practices in research and scholarship. Scoping reviews specifically can outline strengths and weaknesses, and provide insights that can help to advance a field of research, inform future research agendas, and lay the groundwork for other evidence synthesis [13]. Also important, a scoping such as this one can help meet the demand and build the necessary capacity for expert knowledge synthesis that occurs outside of healthcare disciplines [14].

## Methods

While informed by Arksey and O’Malley’s [11] framework and Grant and Booth’s [15] influential work on evidence synthesis reviews, we draw heavily on recent methodological guidance on reviews from the *JBI Manual for Evidence Synthesis* [16] and the *Cochrane Handbook for Systematic Reviews of Interventions* [17], and incorporate considerations raised by Peters et al. [14] The design of this scoping review protocol document is our own, but is informed by Ghezzi-Kopel and Porciello’s [18] open evidence synthesis protocol template.

We have tested initial literature search strategies and further refined and enhanced them using the database thesaurus features found in both Education Resources Information Center (ERIC) and Education Research Complete. These will be tested further before data collection and analysis begins. For our sample frame we have identified relevant information sources (e.g., disciplinary and multidisciplinary databases) to collect publications and which allow the appropriate search limits we have identified (journal publication, etc.). In alignment with the research questions, data collection will focus on finding and comprehensively compiling relevant publications, and all results will be exported to Covidence for screening.

Our team will use Covidence software during the project to conduct deduplication, screening, coding, and data extraction. Covidence provides data management support in the scoping process and helps generate inter-rater reliability scores, as well as a PRISMA flow diagram for reporting. Data analysis of items will be done independently by two researchers coding in parallel, iterative rounds. Final synthesis will focus on overall trends and key findings, as we transition to reporting and dissemination. Review reporting will use the Preferred Reporting Items for Systematic reviews and Meta-Analyses Extension for Scoping Reviews (PRISMA-ScR) [19], which is integrated within Covidence research software.

### Protocol registration

This protocol is preregistered on OSF, available at: https://doi.org/10.17605/OSF.IO/V5WF7.

## Research questions

The research questions guiding our inquiry around social media use in undergraduate settings are:

- RQ1: What methods have been commonly used to study social media technologies in undergraduate teaching and learning over time?
- RQ2: Are there observable trends, patterns, or gaps that can be identified in the existing published literature?

## Objectives

The goal of our scoping review is to address a need to analyse the body of research on the use of social media across undergraduate teaching and learning contexts by identifying notable patterns and themes within studies published in scholarly journals. To understand the state of research in the field, our objectives include determining what methods have been commonly used to study social media in undergraduate learning, and whether there are observable trends, patterns, or gaps with regard to research methods used, data types and sources, the most popular types of social media platforms used in learning, and overall findings in the existing literature, as well as any pandemic learning context insights that emerge. In scoping and synthesising the research on social media in undergraduate settings, we will also make meta-research contributions by disseminating on both our review methods and approaches (as reflected, for example, in our protocol) and the results of the scoping review itself.

## Definitions

The following describes in detail the key definitions that are used within the context of our research.

### Social media

Based on their comprehensive analysis, McCay-Peet and Quan-Haase conclude that there is relative consensus regarding the meaning of social media and define this term as follows: “Social media are web-based services that allow individuals, communities, and organisations to collaborate, connect, interact, and build community by enabling them to create, co-create, modifies [sic], share, and engage with user-generated content that is easily accessible” [20 p17]. This definition of social media has been used in other recent scoping studies [21]. Additionally, in their recent scoping review protocol, Giroux et al. point out that the term social media “refers to any web-based service that allows individuals to construct a public or semi-public profile and interact with other users within a bounded system” [22 pp1-2]. These defining elements, as well as the categories and characteristics outlined by Smith [23], are used to guide our research and search strategies.

*Social media* is also a broad term describing a fluid, evolving suite of technologies. These affordances are influenced by several driving factors and features, including user demand, commercialization and monetization opportunities, and security and privacy concerns. As such, there is ongoing debate about the precise tools and platforms that are considered to be social media, and in light of this we have intentionally delineated the social media technologies that are within the scope of our research through our inclusion and exclusion criteria. Using these definitions, we deemed certain technologies (e.g., text messaging, email, email lists (listservs), and conferencing software) that typically do not focus on sharing and engaging in interactions via a public/semi-public profile to be out of scope. We also deemed platforms focused primarily on virtual worlds to be out of scope.

In higher education learning contexts, common learning management systems (e.g., Moodle, D2L) may embed social media-like elements, but post-secondary institutions usually control the profile capabilities of faculty and students in unique ways, where users have little (if any) choice regarding their accounts and usernames, and otherwise have limited abilities within the system, and these LMS profiles and activities are typically not public or semi-public. As such, we have excluded studies that focus on the LMS or other aspects of online course management and delivery (e.g., MOOCs).

To identify prominently used social media platforms we examined several online sources and reports on social media usage globally and over time [24–26]. and Wikipedia’s *Timeline of Social Media* [27]. We also sought out platforms with the highest number of monthly active users (MAUs) [28–32] and consulted reports on social media platform usage from different sources including an analysis from Newsweek [33], *The State of Social Media in Canada* reports [34–36], the *Social Media Fact Sheet* [37], and a social media use analysis by the *Our World in Data* project [38]. The platforms named in our final list of specific social media to include in the search strategy aligns with our inclusion criteria, while platforms that do not align with the research questions or the study’s focus (e.g., apps specifically for dating) are out of scope. These platform names will be searched for alongside general keyword terms describing social media and social networking (Table 1).

**Table 1:** List of named social media platforms (alphabetically).

4chan	Gettr	Open Diary	Telegram
8chan	Google+	Orkut	TikTok
AmIHotorNot.com (aka Hot or Not, HOTorNOT)	Google Hangouts	Path	Triller
AOL Instant Messenger	Hi5	Patreon	Truth Social
Classmates.com	ICQ	Periscope	Tumblr
Clubhouse	Instagram	Photobucket	Twitch
Co-Star	Justin.tv	Pinterest	Twitter
Bebo	Keek	Parler	VK (aka VKontakte)
Beme	Kuaishou	Pillowfort	Vine
BeReal	LinkedIn	QQ	WeChat
Bolt (aka Bolt.com)	LiveJournal	Quora	Weibo
del.ici.ous (aka Delicious)	LunarStorm	Qzone	Whatsapp
Discord	Mastodon	Reddit	Wikipedia
Douyin	Meerkat	Renren	Windows Live Messenger (aka Windows Messenger)
Facebook (incl.Facebook Messenger)	mIRC	SixDegrees.com	Wordpress
Flickr	MSN Messenger	Skype	XING
FourSquare	Musical.ly	Slack	Yahoo! Messenger
Friendster	Myspace	Snapchat	YikYak
FriendFeed	Nasza Klasa (aka NK.pl)	StumbleUpon	YouTube
Gab	Nexopia	Tagged	

### Undergraduate

As our study focuses on undergraduate teaching and learning it is also important to define what this means. At post-secondary institutions an undergraduate program usually “refers to a program of study that you can pursue immediately after high school—or any time thereafter” [39]. Undergraduate students typically complete these programs at institutions that are often described using different terms: colleges, universities, polytechnics, tertiaries, post-secondaries, and others. Our use of *undergraduates* or *undergraduate students* means the students engaged in learning at these types of institutions while studying toward a degree or credential at a bachelor’s or similar level.

## Eligibility criteria

The following inclusion and exclusion criteria help define this review.

### Inclusion criteria

To define key elements required for our scoping review, including the population, context, and concept (PCC) related to the purpose of the review, we have drawn on guidance [40] within the *JBI Manual for Evidence Synthesis* when defining our inclusion criteria. Items to be included will meet the following criteria:

1. Population: The primary individuals involved in undergraduate teaching and learning including both the students (learners) and the educators (faculty).
2. Concept: The use of social media that meet the definitions above, such as the social media platforms previously mentioned, for the purposes of formal and informal undergraduate teaching and learning.
3. Context: Post-secondary teaching or learning environments such as universities and colleges, including online versions of those environments.
4. Peer-reviewed articles including original theoretical or empirical research, meta-analyses and systematic reviews, case studies, or other quantitative, qualitative, mixed-methods, and experimental research;
5. Articles must be published in an identifiable academic or scholarly journal;
6. Language: English publications only;

We include English-language publications only due to the language fluency of the researchers, while non-English articles will be removed during screening.

### Exclusion criteria

1. Those individuals outside of undergraduate teaching and learning settings (e.g., graduate students, elementary and secondary students, mixed populations), or teaching or learning outside of educational settings that are not part of an undergraduate post-secondary context; university/college administrators or non-academic staff fall into this categorization.
2. Studies where the primary activity is beyond the focus of the research questions, or where the tool or technology used does not meet the above definition and criteria for social media use for higher education purposes, such as use of online dating apps.
3. Teaching or learning activities occurring within a learning management system (LMS) or MOOC, as well as those activities focused on text messaging, email, email lists (listservs), online conferencing, and virtual world interactions.
4. Non-empirical and non-peer-reviewed works, and works situated as opinion-based commentaries, viewpoints, editorials, and perspective pieces, etc.
5. Books, book reviews, and book chapters, and grey literature including government reports, dissertations/theses, conference abstracts, websites, environmental scans, etc.;
6. Language: Published in a language other than English;

Regarding the exclusion of grey literature, the research team understands the potential to introduce risk of bias and otherwise limit this study, and acknowledges that including grey literature is usually desired or required for evidence syntheses. However, we have made this decision to balance feasibility and practicality while still meeting the stated research objective of examining the scholarship published in journals over time.

## Information sources

### Primary databases and indices

We will conduct systematic searching in relevant subject and multidisciplinary databases to find the published research relating to our primary research questions. For this study the primary disciplinary databases are Australian Education Index (Proquest), British Education Index (EBSCO), Education Database (ProQuest), Education Research Complete (EBSCO), and Education Resources Information Center (ERIC) (EBSCO). These will be complemented with searching in the multidisciplinary databases Academic Search Complete (EBSCO) and Scopus (Elsevier).

## Search strategy

The search strategy addresses the PCC framework across three primary categories of search strings. The first focuses on the social media concept using terms and the names of popular platforms, and was derived through a combination of the definitions already provided, the subject-matter expertise of a project researcher (EES), and by reviewing existing, recent literature on the topic including a recent systematic review and related protocol on social media in healthcare [41], as well as several rounds of testing. We also consulted an open search hedge published by an expert librarian [42]. However, given the specific nature of our scoping project and associated research questions, we made significant modifications and expanded the included terms to meet the needs and scope of the social media technologies and related concepts appropriate for our study.

The concepts in the second and third categories are related and have conceptual overlap in their subject matter, but are treated separately within the overall strategy searching. One of these focuses on identifying post-secondary environments (e.g., universities, colleges) and also the population (e.g., students, educators) primarily involved in undergraduate teaching and learning. The third category captures the relevant processes and activities of learning and teaching. These categories are likewise derived from the definitions already provided, as well as researchers’ *a priori* knowledge of, and professional work experience in, educational research, post-secondary education settings, and undergraduate teaching and learning. Terms here were further informed through consultation with a librarian with research experience in social media and the scholarship of teaching and learning in undergraduate contexts.

For all three groups, test searches and use of database thesauri in both ERIC and Education Research Complete ensured identification of relevant indexing terms and descriptors. As one of the researchers for this project is an academic librarian (RH), the development and testing of initial search stings was completed within the research team. The draft strategy has been rigorously tested within ERIC and documented (see S1 Appendix), including the search syntax and the limits used to isolate records indexed as journal article publications. Previously identified seed articles from within ERIC that met all of the inclusion criteria helped verify the search strategy, as they were confirmed to be within the results. (These seed articles were later used to pilot test the data extraction elements.) Earlier versions of the search strategy were reviewed by an independent research librarian using the Peer Review of Electronic Search Strategies (PRESS) guidelines [43] and the suggested revisions have been incorporated into the search strategy as reported in this protocol.

We will identify published research on the scoping project topics through systematic searching in each of the identified sources, using unique strategies for each information source translated to use the appropriate descriptors, syntax, and filters in those resources. All searches will be logged using a spreadsheet in the shared team Google Drive, and search histories saved using the account features within each information source. Records of search strings and exports of search results (citation records) from each information source will be archived and stored in a shared Google Drive accessible to all team members.

## Study records

Researchers will use Covidence review software (https://.covidence.org/) for managing the project and information sources, including importing citations, title/abstract and full-text screening, data extraction, and overall reference management. Deduplication will be done within Covidence’s embedded processes, and any records flagged will be manually reviewed to confirm their duplication.

During the pilot screening phase, researchers will test the inclusion and exclusion criteria against the titles and abstracts using a random selection of 50 records. The team will meet following the pilot activity to discuss discrepancies. Additional rounds of pilot testing will be conducted until the team reaches consensus agreement of 90% or higher. Only then will records will then be screened for inclusion eligibility by the research team in two independent, parallel rounds, with a first phase focusing on titles and abstracts only, and a second phase focusing on full-text of articles. Again, this process will be managed within Covidence. The results of the entire screening process and reasons for exclusions during the full-text screening will be captured and reported using a PRISMA flow diagram.

Much of the data management will occur within Covidence, with some file storage in a shared Google Drive accessible by all team members. Files will follow a standardised naming convention (e.g., databasename_string, databasename_searchdate). Where appropriate these and other records will be shared openly and accessibly in a data repository (or similar) upon completion of the project.

## Data extraction

Piloting of the data extraction elements was completed independently by two researchers reviewing and testing against several seed articles identified while piloting the search strategy.

Data extraction will occur using the extraction processes in Covidence. The extraction elements have been selected to focus on the research questions and inclusion criteria (including PCC). These may be updated or revised during extraction; for example, to include emergent themes or previously unidentified tools or trends. These iterative changes or modifications will be recorded and described in detail within the review manuscript. The following elements will be captured via the data extraction process:

Data extraction elements

- Citation details (e.g., author/s, date, title, journal, volume, issue, pages)
- Country (or countries) where the research took place
- Purpose/objectives of the research
- Research method(s) (e.g., survey, case study, interviews, etc.)
- Research data type/sources (e.g., qualitative, quantitative, mixed methods, etc.)
- Specific social media platform(s) mentioned (e.g., Facebook, Twitter, Instagram, etc.)
- Is the primary focus on teaching or learning (teaching focus, learning focus, approximately equal, or undefined)?
- Does the study focus on the COVID-19 pandemic or emergency remote teaching or learning?
- Key findings/results of the research

Data extraction from each source will be conducted independently by (at least) two researchers who will complete their reviews within Covidence. Discrepancies that arise between reviewers will be resolved through discussion to reach consensus; this stage will also be used to address coding errors or missing values. For records where consensus cannot be reached, a third researcher will review to make a final decision.

## Data synthesis and charting

Data will be presented and reported using tabular, numeric, and/or graphical formats to represent the extracted data elements described above. These will be accompanied by a narrative summary addressing the research questions guiding this project. These will focus on an analysis of the research methods commonly used to study social media technologies in undergraduate settings, and include any key findings, trends, patterns, or gaps identified in the existing research literature relating to social media and teaching and learning. Reporting of search strategy results and other elements will follow PRISMA-ScR standards [19].

### Dissemination plans

Results will be shared via appropriate academic conferences, and via a journal article submitted to an academic journal. We also anticipate disseminating non-traditional knowledge translation artefacts (e.g., infographics) and making further meta-research contributions.

## Supporting information

S1 Appendix Search strategy draft using ERIC (via Ebsco). (DOCX)

S2 Appendix PRISMA-P checklist. (DOCX)
