# Supplementary material for: Social media in undergraduate teaching and learning: A scoping review protocol
Source: PLoS One. 2023 Nov 28;18(11):e0291306. doi: 10.1371/journal.pone.0291306 (PMC10684007; doi:10.1371/journal.pone.0291306)
Supplement: S1 Appendix — (DOCX) [file pone.0291306.s001.docx]

### **S1 Appendix. Search strategy draft using ERIC (via Ebsco).**

| **#** | **Query** | **Search Options** | **Results** |
| --- | --- | --- | --- |
| S1 | DE "Social Media" OR DE "Social Networks" OR DE "Web 2.0 Technologies" OR DE "Synchronous Communication"  DE "Discussion Groups" | Boolean/Phrase | 15,390 |
| S2 | TI ( (social W0 (media* OR network* OR software* OR news* OR bookmark* OR platform* OR web* OR stream* OR site* OR app OR apps OR application*)) OR ((video OR media OR online OR file OR image OR photo) W0 shar*) OR ((video OR web) N1 (stream* OR cast*)) OR ((online OR web OR virtual OR digital) N0 (discussion* OR messag* OR board* OR forum* OR feed*)) OR (blog* OR weblog*) OR (podcast* OR vodcast* OR webcast*) OR ((user OR crowd*) W0 (generated OR created OR sourced) W1 content)) OR ((chat* N0 (group* OR online OR room* OR instant OR internet)) OR "chatroom*" OR IRC) OR ((internet OR online OR virtual) N1 communit*) OR ("really simple syndication" OR RSS OR newsfeed*) OR ((online OR web) W0 (professional OR academic) W1 network*) OR wiki* OR tweet* ) OR AB ( (social W0 (media* OR network* OR software* OR news* OR bookmark* OR platform* OR web* OR stream* OR site* OR app OR apps OR application)) OR ((video OR media OR online OR file OR image OR photo) W0 shar*) OR ((video OR web) N1 (stream* OR cast*)) OR ((online OR web OR virtual OR digital) N0 (discussion* OR messag* OR board* OR forum* OR feed*)) OR (blog* OR weblog*) OR (podcast* OR vodcast* OR webcast*) OR ((user OR crowd*) W0 (generated OR created OR sourced) W1 content)) OR ((chat* N0 (group* OR online OR room* OR instant OR internet)) OR "chatroom*" OR IRC) OR ((internet OR online OR virtual) N1 communit*) OR ("really simple syndication" OR RSS OR newsfeed*) OR ((online OR web) W0 (professional OR academic) W1 network*) OR wiki* OR tweet* ) OR KW ( (social W0 (media* OR network* OR software* OR news* OR bookmark* OR platform* OR web* OR stream* OR site* OR app OR apps OR application*)) OR ((video OR media OR online OR file OR image OR photo) W0 shar*) OR ((video OR web) N1 (stream* OR cast*)) OR ((online OR web OR virtual OR digital) N0 (discussion* OR messag* OR board* OR forum* OR feed*)) OR (blog* OR weblog*) OR (podcast* OR vodcast* OR webcast*) OR ((user OR crowd*) W0 (generated OR created OR sourced) W1 content)) OR ((chat* N0 (group* OR online OR room* OR instant OR internet)) OR "chatroom*" OR IRC) OR ((internet OR online OR virtual) N1 communit*) OR ("really simple syndication" OR RSS OR newsfeed*) OR ((online OR web) W0 (professional OR academic) W1 network*) OR wiki* OR tweet* ) | Boolean/Phrase | 19,402 |
| S3 | TI ( 4chan OR 8chan OR ("AmIHotorNot.com" OR “HOTorNOT”) OR ("AOL Instant Messenger" OR (AOL AND AIM)) OR “Classmates.com” OR Bebo OR BeReal OR “Bolt.com” OR "del.ici.ous" OR Douyin OR (Facebook OR "Facebook Messenger") OR Flickr OR FourSquare OR Friendster OR FriendFeed OR Gettr OR ("Google+" OR "Google Plus") OR "Google Hangouts" OR Hi5 OR ICQ OR Instagram OR "Justin.tv" OR Keek OR Kuaishou OR LinkedIn OR LiveJournal OR LunarStorm OR mIRC OR "MSN Messenger" OR "Musical.ly" OR Myspace OR ("Nasza Klasa" OR “NK.pl”) OR Nexopia OR "Open Diary" OR Orkut OR Patreon OR Photobucket OR Pinterest OR Pillowfort OR Quora OR Qzone OR Reddit OR Renren OR "SixDegrees.com" OR Skype OR Snapchat OR StumbleUpon OR TikTok OR Triller OR "Truth Social" OR Tumblr OR Twitter OR (VK OR VKontakte) OR WeChat OR Weibo OR Whatsapp OR Wikipedia OR ("Windows Live Messenger" OR "Windows Messenger") OR Wordpress OR XING OR "Yahoo! Messenger" OR "Yik Yak" OR YouTube OR ((Clubhouse OR Beme OR Bolt OR “Co-Star” OR Delicious OR Discord OR Gab OR “Hot or Not” OR Mastodon OR Meerkat OR Path OR Parler OR Periscope OR QQ OR Slack OR Tagged OR Telegram OR Twitch OR Vine) N1 (platform* OR social OR software OR technolog* OR app OR apps OR application OR network* OR messag*)) ) OR AB ( 4chan OR 8chan OR ("AmIHotorNot.com" OR “HOTorNOT”) OR ("AOL Instant Messenger" OR (AOL AND AIM)) OR “Classmates.com” OR Bebo OR BeReal OR “Bolt.com” OR "del.ici.ous" OR Douyin OR (Facebook OR "Facebook Messenger") OR Flickr OR FourSquare OR Friendster OR FriendFeed OR Gettr OR ("Google+" OR "Google Plus") OR "Google Hangouts" OR Hi5 OR ICQ OR Instagram OR "Justin.tv" OR Keek OR Kuaishou OR LinkedIn OR LiveJournal OR LunarStorm OR mIRC OR "MSN Messenger" OR "Musical.ly" OR Myspace OR ("Nasza Klasa" OR “NK.pl”) OR Nexopia OR "Open Diary" OR Orkut OR Patreon OR Photobucket OR Pinterest OR Pillowfort OR Quora OR Qzone OR Reddit OR Renren OR "SixDegrees.com" OR Skype OR Snapchat OR StumbleUpon OR TikTok OR Triller OR "Truth Social" OR Tumblr OR Twitter OR (VK OR VKontakte) OR WeChat OR Weibo OR Whatsapp OR Wikipedia OR ("Windows Live Messenger" OR "Windows Messenger") OR Wordpress OR XING OR "Yahoo! Messenger" OR "Yik Yak" OR YouTube OR ((Clubhouse OR Beme OR Bolt OR “Co-Star” OR Delicious OR Discord OR Gab OR “Hot or Not” OR Mastodon OR Meerkat OR Path OR Parler OR Periscope OR QQ OR Slack OR Tagged OR Telegram OR Twitch OR Vine) N1 (platform* OR social OR software OR technolog* OR app OR apps OR application OR network* OR messag*)) ) OR KW ( 4chan OR 8chan OR ("AmIHotorNot.com" OR “HOTorNOT”) OR ("AOL Instant Messenger" OR (AOL AND AIM)) OR “Classmates.com” OR Bebo OR BeReal OR “Bolt.com” OR "del.ici.ous" OR Douyin OR (Facebook OR "Facebook Messenger") OR Flickr OR FourSquare OR Friendster OR FriendFeed OR Gettr OR ("Google+" OR "Google Plus") OR "Google Hangouts" OR Hi5 OR ICQ OR Instagram OR "Justin.tv" OR Keek OR Kuaishou OR LinkedIn OR LiveJournal OR LunarStorm OR mIRC OR "MSN Messenger" OR "Musical.ly" OR Myspace OR ("Nasza Klasa" OR “NK.pl”) OR Nexopia OR "Open Diary" OR Orkut OR Patreon OR Photobucket OR Pinterest OR Pillowfort OR Quora OR Qzone OR Reddit OR Renren OR "SixDegrees.com" OR Skype OR Snapchat OR StumbleUpon OR TikTok OR Triller OR "Truth Social" OR Tumblr OR Twitter OR (VK OR VKontakte) OR WeChat OR Weibo OR Whatsapp OR Wikipedia OR ("Windows Live Messenger" OR "Windows Messenger") OR Wordpress OR XING OR "Yahoo! Messenger" OR "Yik Yak" OR YouTube OR ((Clubhouse OR Beme OR Bolt OR “Co-Star” OR Delicious OR Discord OR Gab OR “Hot or Not” OR Mastodon OR Meerkat OR Path OR Parler OR Periscope OR QQ OR Slack OR Tagged OR Telegram OR Twitch OR Vine) N1 (platform* OR social OR software OR technolog* OR app OR apps OR application OR network* OR messag*)) ) |  | 4,991 |
| S4 | S1 OR S2 OR S3 | Boolean/Phrase | 26,996 |
| S5 | DE "Academic Education" OR DE "College Environment" OR DE "Colleges" OR DE "Community Colleges" OR DE "Classroom Environment" OR DE "Educational Environment" OR DE "Experimental Colleges" OR DE "Higher Education" OR DE "Land Grant Universities" OR DE "Multicampus Colleges" OR "Noncampus Colleges" DE "Online Courses" OR DE "Open Universities" OR DE "Postsecondary Education" OR DE "Private Colleges" OR DE "Public Colleges" OR DE "Research Universities" OR DE "Small Colleges" OR DE "State Colleges" OR DE "State Schools" OR DE "State Universities" OR DE "Technical Institutes" OR DE "Two Year Colleges" OR DE "Universities" OR DE "Upper Division Colleges" OR DE "Urban Universities" OR DE "Virtual Classrooms" OR DE "Virtual Schools" OR DE "Virtual Universities" OR DE "Vocational Education" OR DE "Vocational Schools" OR DE "Adult Educators" OR DE "Adult Students" OR DE "College Faculty" OR DE "College Freshmen" OR DE "College Seniors" OR DE "College Students" OR DE "Faculty" OR DE "Teachers" OR DE "Trainees" OR DE "Trainers" OR DE "Two Year College Students" OR DE "Undergraduate Students" OR DE "Undergraduate Study" | Boolean/Phrase | 683,609 |
| S6 | EL “Adult Education” OR EL “Higher Education” OR EL “Postsecondary Education” OR EL “Two Year Colleges” | Boolean/Phrase | 291,080 |
| S7 | TI ( universit* OR colleg* OR post-secondar* OR postsecondar* OR "higher education" OR cegep* OR ((polytechnic* OR academ* OR vocational* OR tertiary) N1 (education OR institution* OR setting* OR school* OR student*)) OR undergrad* OR freshm?n* OR sophomore* OR junior* OR senior* OR baccalaureate* OR bachelor* OR trainer* OR trainee* OR fellow* OR faculty OR professor* OR lecturer* OR instructor* ) OR AB ( universit* OR colleg* OR post-secondar* OR postsecondar* OR "higher education" OR cegep* OR ((polytechnic* OR academ* OR vocational* OR tertiary) N1 (education OR institution* OR setting* OR school* OR student*)) OR undergrad* OR freshm?n* OR sophomore* OR junior* OR senior* OR baccalaureate* OR bachelor* OR trainer* OR trainee* OR fellow* OR faculty OR professor* OR lecturer* OR instructor* ) OR KW ( universit* OR colleg* OR post-secondar* OR postsecondar* OR "higher education" OR cegep* OR ((polytechnic* OR academ* OR vocational* OR tertiary) N1 (education OR institution* OR setting* OR school* OR student*)) OR undergrad* OR freshm?n* OR sophomore* OR junior* OR senior* OR baccalaureate* OR bachelor* OR trainer* OR trainee* OR fellow* OR faculty OR professor* OR lecturer* OR instructor* ) | Boolean/Phrase | 571,816 |
| S8 | S5 OR S6 OR S7 | Boolean/Phrase | 822,944 |
| S9 | DE "Learning" OR DE "Active Learning" OR DE "Adult Learning" OR DE "Authentic Learning" OR DE "Cooperative Learning" OR DE "Discovery Learning" OR DE "Electronic Learning" OR DE "Experiential Learning" OR DE "Problem Based Learning" OR DE "Situated Learning" OR DE "Student Centered Learning" OR DE "Transformative Learning" OR DE "Instruction" OR DE "College Instruction" OR DE "Andragogy" OR DE "Training" OR DE "Study" OR DE "Learning Activities" OR DE "Learning Experience" OR DE "Learner Engagement" OR DE "Inquiry” OR DE "Technology Uses in Education" OR DE "Computer Assisted Instruction" OR DE "Web Based Instruction" OR DE "Blended Learning" OR DE "Technology Integration" OR DE "Educational Technology" OR DE "Distance Education" OR DE "Instructional Innovation" OR DE "Educational Innovation" OR DE "Influence of Technology" | Boolean/Phrase | 296,665 |
| S10 | TI ( teaching OR teach OR learning OR learn OR educati* OR instructi* OR training OR pedagog* OR andragog* OR e-learning or "electronic learning” OR inquiry OR enquiry ) OR AB ( teaching OR learning OR educati* OR instructi* OR training OR pedagog* OR andragog* OR e-learning or "electronic learning” OR inquiry OR enquiry ) OR KW ( teaching OR learning OR educati* OR instructi* OR training OR pedagog* OR andragog* OR e-learning or "electronic learning” OR inquiry OR enquiry ) | Boolean/Phrase | 1,193,181 |
| S11 | S9 OR S10 | Boolean/Phrase | 1,232,033 |
| S12 | S4 AND S8 AND S11 | Boolean/Phrase | 13,598 |
| S13 | S12 | Limiters - Publication Type: Journal Articles  Search modes - Boolean/Phrase | 10,905 |

Note: This search was documented on 22 June 2023.
